# Crystal structure and Hirshfeld surface analysis of 1-[6-bromo-2-(3-bromo­phen­yl)-1,2,3,4-tetra­hydro­quinolin-4-yl]pyrrolidin-2-one

**DOI:** 10.1107/S2056989024008144

**Published:** 2024-08-30

**Authors:** Anastasia A. Pronina, Alexandra G. Kutasevich, Mikhail S. Grigoriev, Khudayar I. Hasanov, Nurlana D. Sadikhova, Tahir A. Javadzade, Mehmet Akkurt, Ajaya Bhattarai

**Affiliations:** a RUDN University, 6 Miklukho-Maklaya St, Moscow, 117198, Russian Federation; b Frumkin Institute of Physical Chemistry and Electrochemistry, Russian Academy of Sciences, Leninskiy prospect 31-4, Moscow 119071, Russian Federation; c Western Caspian University, Istiqlaliyyat Street 31, AZ1001, Baku, Azerbaijan; d Azerbaijan Medical University, Scientific Research Centre (SRC), A. Kasumzade St. 14. AZ 1022, Baku, Azerbaijan; e Department of Chemistry, Baku State University, Z. Xalilov Str. 23, Az 1148 Baku, Azerbaijan; f Department of Chemistry and Chemical Engineering, Khazar University, Mahsati St. 41, AZ 1096, Baku, Azerbaijan; g Department of Physics, Faculty of Sciences, Erciyes University, 38039 Kayseri, Türkiye; h Department of Chemistry, M.M.A.M.C (Tribhuvan University) Biratnagar, Nepal; Institute of Chemistry, Chinese Academy of Sciences

**Keywords:** crystal structure, hydrogen bonds, the Povarov method, tetra­hydro­quinoline, Hirshfeld surface analysis

## Abstract

This study presents the synthesis, characterization and Hirshfeld surface analysis of 1-[6-bromo-2-(3-bromo­phen­yl)-1,2,3,4-tetra­hydro­quinolin-4-yl]pyrrolidin-2-one, C_19_H_18_Br_2_N_2_O. In the title compound, the pyrrolidine ring adopts a distorted envelope configuration. In the crystal, mol­ecules are linked by inter­molecular N—H⋯O, C—H⋯O and C—H⋯Br hydrogen bonds, forming a three-dimensional network. In addition, pairs of mol­ecules along the *c* axis are connected by C—H⋯π inter­actions. According to a Hirshfeld surface study, H⋯H (36.9%), Br⋯H/H⋯Br (28.2%) and C⋯H/H⋯C (24.3%) inter­actions are the most significant contributors to the crystal packing.

## Abstract 2

In the crystal, mol­ecules are linked by inter­molecular N—H⋯O, C—H⋯O and C—H⋯Br hydrogen bonds, forming a three-dimensional network. In addition, pairs of mol­ecules along the *c* axis are connected by C—H⋯π inter­actions.

## 1. Chemical context

Currently, a large number of derivatives of known tetra­hydro­quinolines are promising candidates for testing against various types of biological activity. This class of *N*-heterocyclic compounds has attracted attention of biochemists for the past 50 years, as derivatives of the tetra­hydro­quinoline frame possess anti­bacterial, anti­tumor, and anti­allergic properties. Some are already used as medicinal agents (Ghashghaei *et al.*, 2018 ▸).

In this regard, the synthesis and modification of the tetra­hydro­quinoline system to search for new drugs is an important task in organic chemistry. Over the years, several synthetic routes have been developed to obtain variously substituted tetra­hydro­quinolines (Sridharan *et al.*, 2011 ▸). However, the advantage remains with the Povarov reaction, due to the flexibility of this method, allowing the one-step synthesis of variously substituted 1,2,3,4-tetra­hydro­quinolines (Zubkov *et al.*, 2007 ▸, 2010 ▸; Kouznetsov, 2009 ▸; Varma *et al.*, 2010 ▸; Zaytsev *et al.*, 2013 ▸). Furthermore, the Povarov reaction is characterized by good yields and mild reaction conditions. Usually the reaction proceeds in two stages. The first stage is an aza-Diels–Alder reaction between *N*-aryl­imine and an electron-rich olefin in the presence of catalytic amounts of Lewis acid, which leads to the formation of a cyclo­adduct. The second stage involves a 1,3-H shift in the cyclo­adduct and results in the formation of the tetra­hydro­quinoline moiety.
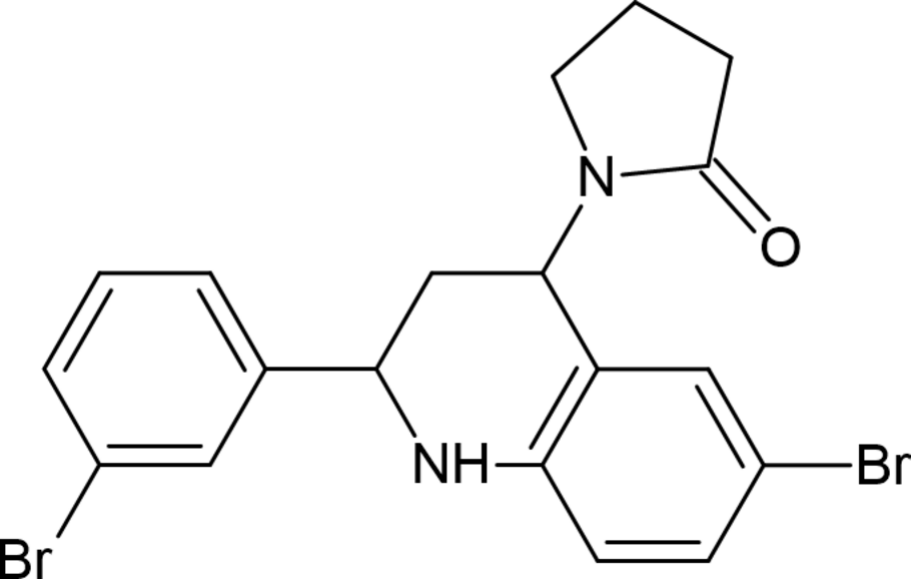


**Figure 1:**
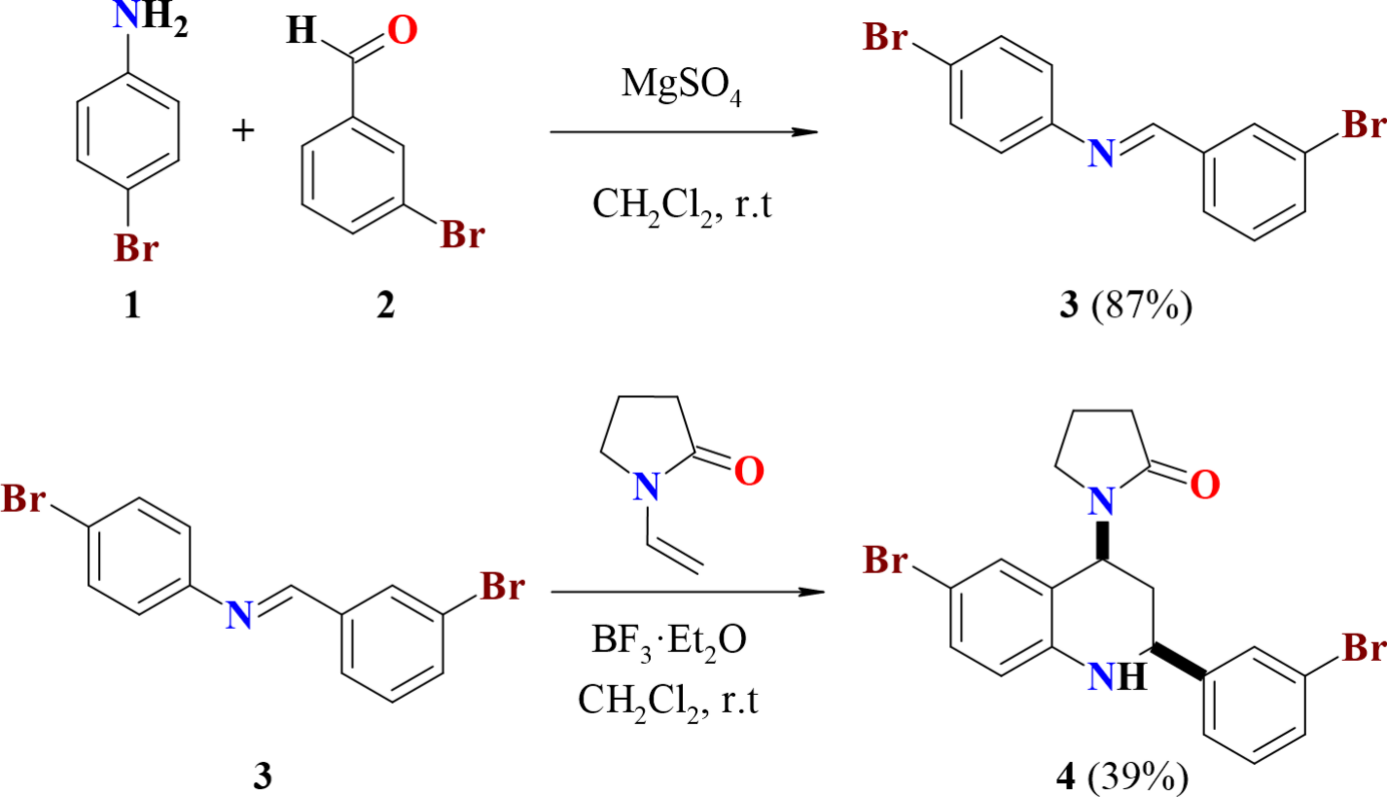
Synthesis of 1-[6-bromo-2-(3-bromo­phen­yl)-1,2,3,4-tetra­hydro­quinolin-4-yl]pyrrolidin-2-one (**4**).

In this work, the synthesis of the corresponding azomethine **3** was carried out using a condensation reaction between 4-bromo­aniline (**1**) and 3-bromo­benzaldehyde (**2**) to form product **3**, which was then introduced into the Povarov reaction. *N*-Vinyl­pyrrolidin-2-one was used as the alkene, and boron trifluoride etherate served as the Lewis acid (Fig. 1 ▸).

Thus, the Povarov method provides a convenient approach for the one-pot synthesis of substituted, partially hydrogenated quinolines and medicinal preparations based on the tetra­hydro­quinoline frame. Some stereochemical features of the resulting adduct **4** are discussed in this work. This work also discusses some stereochemical features of the resulting adduct **4**.

## 2. Structural commentary

**Figure 2:**
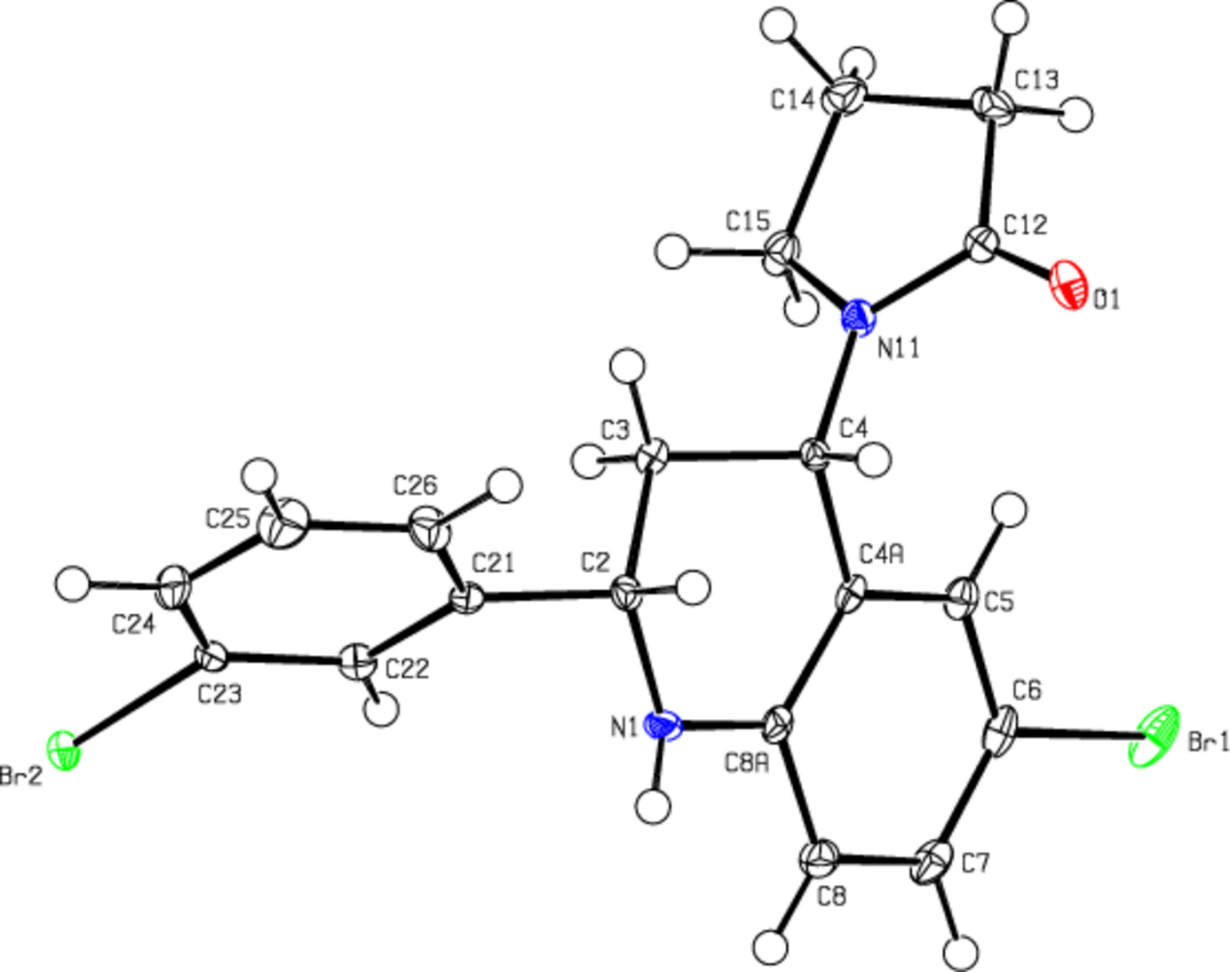
View of the title mol­ecule. Displacement ellipsoids are drawn at the 50% probability level.

In the title compound (Fig. 2 ▸), the 1,2,3,4-tetra­hydro­pyridine ring (N1/C2–C4/C4*A*/C8*A*) of the 1,2,3,4-tetra­hydro­quinoline ring system (N1/C2–C4/C4*A*/C5–C8/C8*A*) adopts an envelope conformation [the puckering parameters (Cremer & Pople, 1975 ▸) are *Q*_T_ = 0.523 (2) Å, θ = 131.7 (2)°, φ = 300.9 (3)°], while the benzene ring (C4*A/*C5–C8/C8*A*) is essentially planar (r.m.s. deviation = 0.002 Å). The plane (r.m.s deviation = 0.002 Å) of the 1,2,3,4-tetra­hydro­quinoline ring system forms angles of 56.85 (9) and 83.05 (10)°, respectively, with the bromo­benzene ring (C21–C26) and the pyrrolidine ring (N11/ C12–C15; r.m.s deviation = 0.002 Å), which has a distorted envelope conformation [the puckering parameters are *Q*(2) = 0.225 (2) Å, φ(2) = 117.2 (6)°]. The angle between the pyrrolidine and bromo­benzene rings is 84.92 (12)°. The geometric parameters in the mol­ecule are normal and in good agreement with those in the compounds discussed in the *Database survey* (section 4).

## 3. Supra­molecular features and Hirshfeld surface analysis

**Figure 3:**
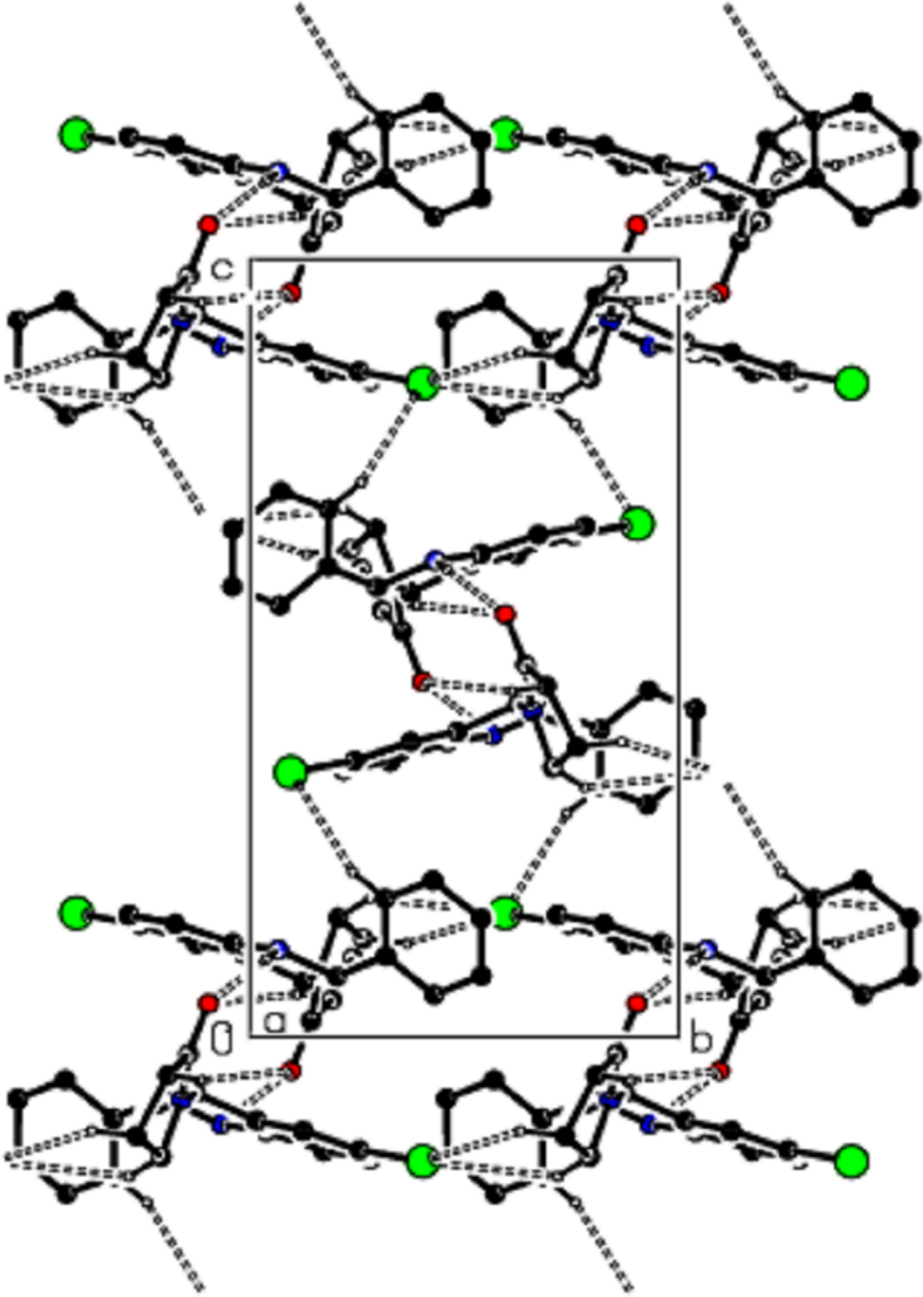
A view of the mol­ecular packing along the *a* axis of the title compound, showing the N—H⋯O, C—H⋯O and C—H⋯Br hydrogen bonds as dashed lines.

**Figure 6:**
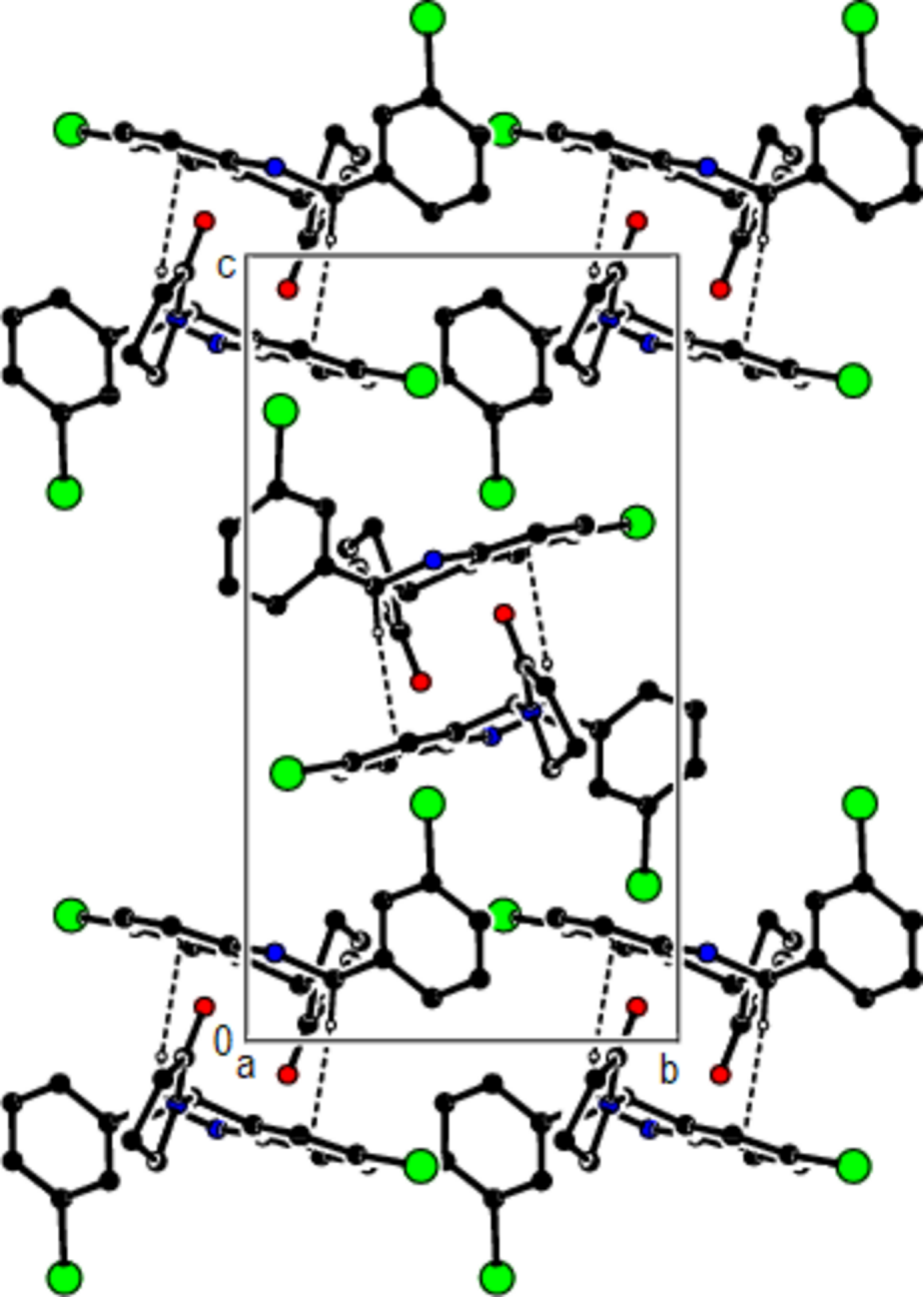
A view of the mol­ecular packing along the *a* axis of the title compound, showing the C—H⋯π inter­actions.

**Figure 9:**
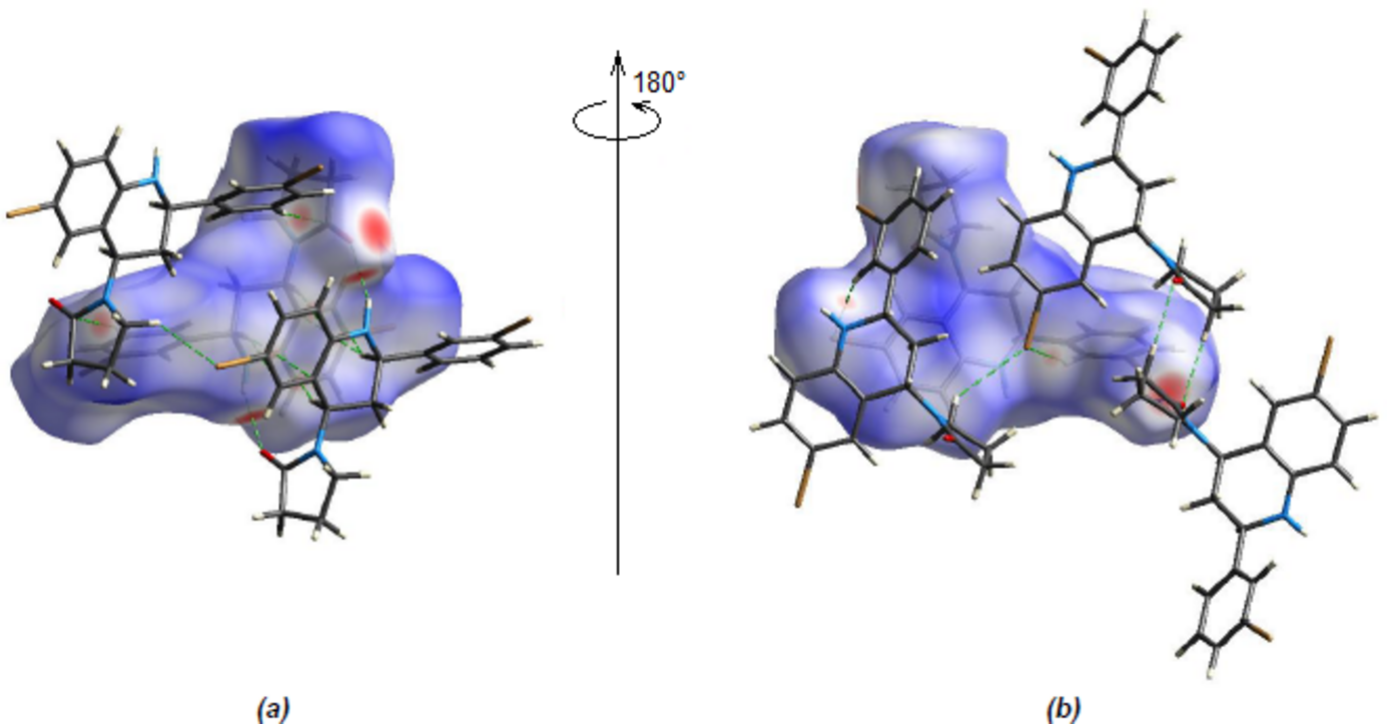
(*a*) Front and (*b*) back views of the three-dimensional Hirshfeld surface for the title compound. Some N—H⋯O, C—H⋯O and C—H⋯Br inter­actions are shown as dashed lines.

**Figure 10:**
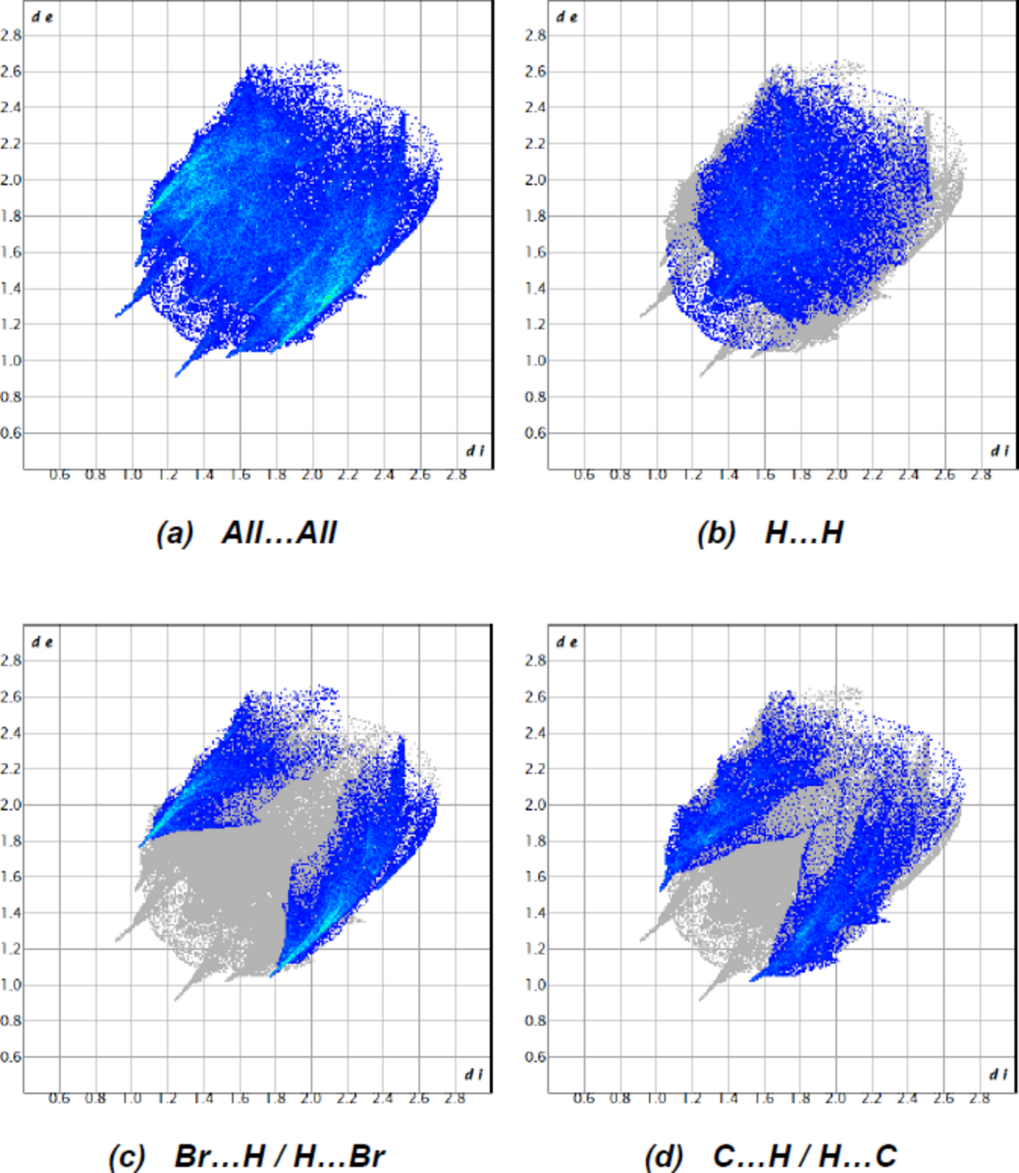
The two-dimensional fingerprint plots for the title compound showing (*a*) all inter­actions, and delineated into (*b*) H⋯H, (*c*) Br⋯H/H⋯Br and (*d*) C⋯H/H⋯C inter­actions. The *d_i_* and *d_e_* values are the closest inter­nal and external distances (in Å) from given points on the Hirshfeld surface.

In the crystal, mol­ecules are linked by inter­molecular N—H⋯O, C—H⋯O and C—H⋯Br hydrogen bonds, forming a three-dimensional network (Table 1 ▸; Figs. 3 ▸, 4 ▸ and 5 ▸). In addition, pairs of mol­ecules along the *c* axis are connected by C—H⋯π inter­actions (Table 1 ▸; Figs. 6 ▸, 7 ▸ and 8 ▸). To qu­antify the inter­molecular inter­actions in the crystal, the Hirshfeld surfaces of the title mol­ecule and the two-dimensional fingerprints were generated with *CrystalExplorer17.5* (Spackman *et al.*, 2021 ▸). On the *d*_norm_ surfaces, bright-red spots show the locations of the N—H⋯O, C—H⋯O and C—H⋯Br inter­actions (Table 1 ▸; Fig. 9 ▸*a*,*b*). The overall two-dimensional fingerprint plot for the title compound and those delineated into H⋯H (Fig. 10 ▸*b*; 36.9%), Br⋯H/H⋯Br (Fig. 10 ▸*c*; 28.2%) and C⋯H/H⋯C (Fig. 10 ▸*d*; 24.3%) contacts are shown in Fig. 10 ▸. O⋯H/H⋯O (7.1%), Br⋯O/O⋯Br (1.8%), Br⋯C/C⋯Br (0.9%), N⋯H/H⋯N (0.4%) and Br⋯N/N⋯Br (0.3%) contacts have little directional influence on the mol­ecular packing.

## 4. Database survey

A search of the Cambridge Structural Database (CSD, Version 5.42, update of September 2021; Groom *et al.*, 2016 ▸) for similar structures with the 1,2,3,4-tetra­hydro­quinoline unit showed that the seven most closely related to the title compound are refcodes POSWAZ (Pronina *et al.*, 2024 ▸), EZOMIR (Çelik *et al.*, 2016 ▸), SUFDEE (Jeyaseelan, *et al.*, 2015*c* ▸), NOVGAI (Jeyaseelan *et al.*, 2015*a* ▸), WUFBEG (Jeyaseelan *et al.*, 2015*b* ▸), WACWOO (Çelik *et al.*, 2010*a* ▸) and CEDNUW (Çelik *et al.*, 2010*b* ▸).

In the crystal of POSWAZ, mol­ecules are linked by inter­molecular N—H⋯O, C—H⋯O, C—H⋯F and C—H⋯Br hydrogen bonds, forming a three-dimensional network. In addition, C—H⋯π inter­actions connect mol­ecules into ribbons along the *b*-axis direction, consolidating the mol­ecular packing. In the crystal of EZOMIR, inversion dimers linked by pairs of N—H⋯N hydrogen bonds generate 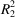
(12) loops. In the crystal of SUFDEE, mol­ecules are linked by weak C—H⋯O hydrogen bonds, generating *C*(8) and *C*(4) chains propagating along [100] and [010], respectively, which together generate (001) sheets. In the crystal of NOVGAI, inversion dimers linked by pairs of C—H⋯O hydrogen bonds generate 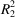
(8) loops. In the crystal of WUFBEG, inversion dimers linked by pairs of C—H⋯O hydrogen bonds generate 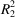
(10) loops. Additional inter­molecular C—H⋯O hydrogen bonds generate *C*(7) chains along [100]. The crystal structure of WACWOO is stabilized by weak aromatic π–π inter­actions [centroid–centroid distance = 3.802 (4) Å] between the pyridine and benzene rings of the quinoline ring systems of adjacent mol­ecules. In the crystal of CEDNUW, π–π stacking inter­actions are present between the pyridine and benzene rings of adjacent mol­ecules [centroid–centroid distances = 3.634 (4) Å], and short Br⋯Br contacts [3.4443 (13) Å] occur.

## 5. Synthesis and crystallization

**N-[(*****E*****)-(3-Bromo­phen­yl)methyl­idene]-4-bromoaniline (3):** A mixture of 4-bromoaniline (**1**) (2.00 g, 0.012 mol), 3-bromobenzaldehyde (**2**) (2.22 g, 0.012 mol) and anhydrous MgSO_4_ (2.89 g, 0.024 mol) was stirred in CH_2_Cl_2_ (40 mL) for 24 h at room temperature. Then, the reaction mixture was passed through a silica gel layer (2 × 3 cm) (eluent CH_2_Cl_2_) and the solvent was evaporated under reduced pressure. Compound **3** was isolated as a yellow powder in 87% yield (3.54 g).

**1-(6-Bromo-2-(3-bromo­phen­yl)-1,2,3,4-tetra­hydro­quinolin-4-yl)pyrrolidin-2-one (4):** Boron trifluoride ether (0.25 mL, 0.002 mol) and *N*-vinyl­pyrrolidin-2-one (1.18 mL, 0.011 mol) were added sequentially to a solution of the azomethine (**3**) (3.5 g, 0.010 mol) in freshly distilled CH_2_Cl_2_ (30 mL), under cooling (275–277 K) and constant stirring. The reaction was monitored by TLC (EtOAc/hexane, 1:2). After the reaction was complete (∼24 h), the reaction mixture was treated with a small amount of water (0.2–0.3 mL) to decompose the catalyst. Then the resulting mixture was passed through a layer of silica gel (2 × 3 cm) and washed with dry CH_2_Cl_2_ (2 × 25 mL). The solvent was evaporated under reduced pressure. The obtained precipitate was recrystallized from a mixture of hexa­ne/EtOAc. The desired product, **4**, was isolated as a white microcrystalline precipitate in 39% yield (1.76 g), m.p. 470.3–471.8 K. IR (KBr), ν (cm^−1^): 3344 (NH), 2951 (Ph), 2889 (Ph), 1667 (N—C=O). ^1^H NMR (700.2 MHz, CDCl_3_, 298 K) (*J*, Hz): *δ* 2.01–2.10 (*m*, 4H, H-3 + H-4-pyrrole), 2.43–2.48 (*m*, 1H, H-3-pyrrole-A), 2.52–2.57 (*m*, 1H, H-3-pyrrole-B), 3.19–3.26 (*m*, 2H, H-5-pyrrole), 4.07 (*s*, 1H, NH), 4.54 (*dd*, *J* = 11.2, *J* = 2.9, 1H, H-2), 5.66 (*dd*, *J* = 11.7, *J* = 6.0 Hz, 1H, H-4), 6.49 (*d*, *J* = 8.6, 1H, H-8), 6.95 (*s*, 1H, H-5), 7.15 (*dd*, *J* = 8.6, *J* = 2.2, 1H, H-7), 7.24 (*t*, *J* = 7.9, 1H, H-5-C_6_H_4_-Br), 7.31 (*d*, *J* = 7.6, 1H, H-6-C_6_H_4_-Br), 7.45 (*d*, *J* = 7.9, 1H, H-4-C_6_H_4_-Br), 7.61 (*s*, 1H, H-2-C_6_H_4_-Br) ppm. ^13^C{^1^H} NMR (176 MHz, CDCl_3_, 298 K): *δ* 18.00, 31.00, 34.58, 42.03, 47.79, 55.60, 109.93, 116.48, 120.73, 122.71, 125.08, 128.93, 129.17, 130.20, 130.95, 131.00, 144.33, 144.69, 175.66 ppm.

Elemental analysis calculated (%) for C_19_H_18_Br_2_N_2_O: C, 50.69; H, 4.03; N, 6.22; found: C, 50.61; H, 3.94; N, 6.42.

Single crystals (splices of prisms) of compound **4** were grown from a mixture of hexane and ethyl acetate (∼3:1).

## 6. Refinement

Crystal data, data collection and structure refinement details are summarized in Table 2 ▸. The C-bound H atoms were placed in calculated positions (0.95–1.00 Å) and refined as riding with *U*_iso_(H) = 1.2*U*_eq_(C). The N-bound H atom was located in a difference map and freely refined.

## Supplementary Material

Crystal structure: contains datablock(s) I. DOI: 10.1107/S2056989024008144/nx2012sup1.cif

Structure factors: contains datablock(s) I. DOI: 10.1107/S2056989024008144/nx2012Isup2.hkl

Supporting information file. DOI: 10.1107/S2056989024008144/nx2012Isup3.cml

CCDC reference: 2378018

Additional supporting information:  crystallographic information; 3D view; checkCIF report

## Figures and Tables

**Figure 4:**
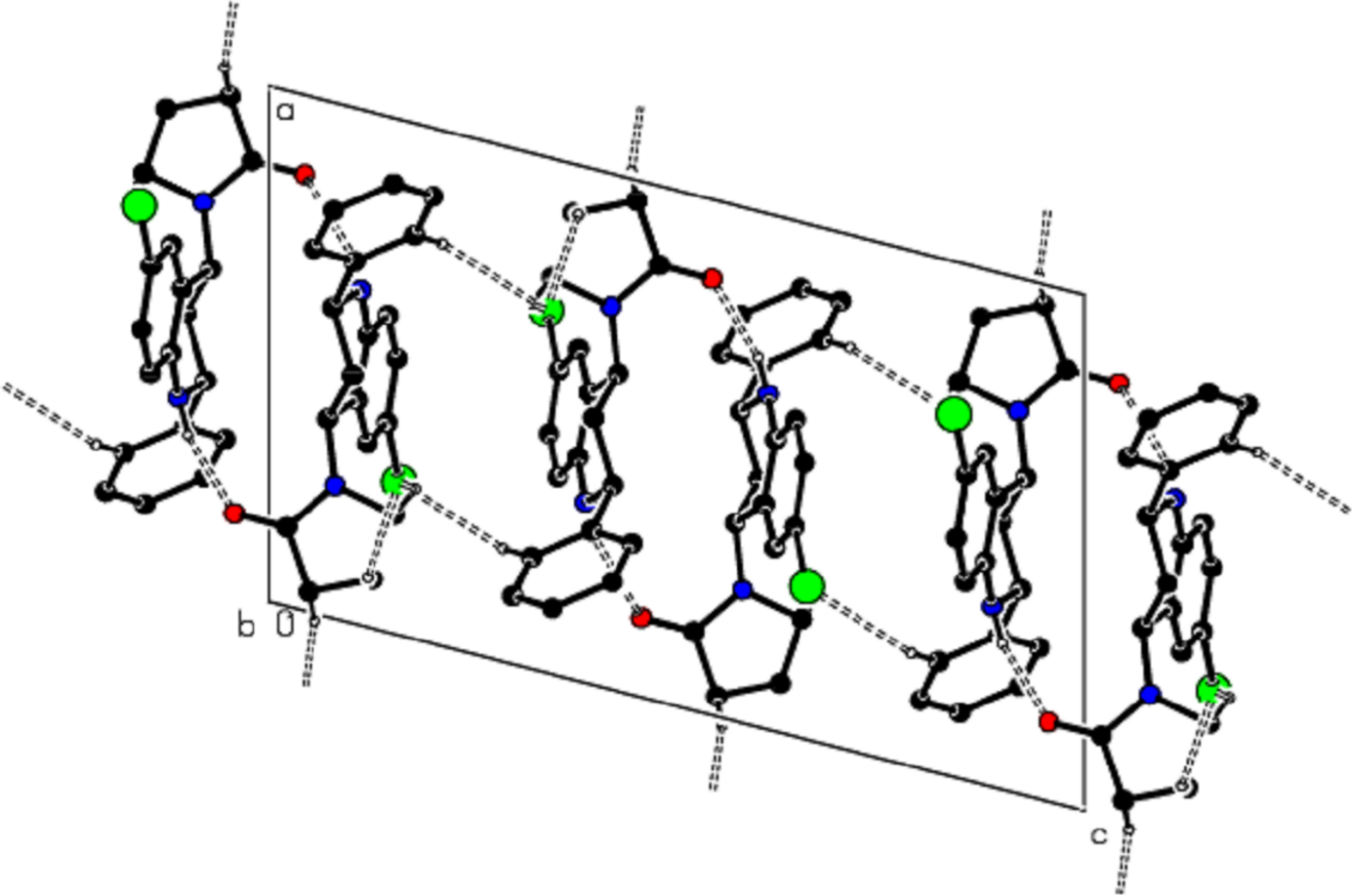
A view of the mol­ecular packing along the *b* axis of the title compound.

**Figure 5:**
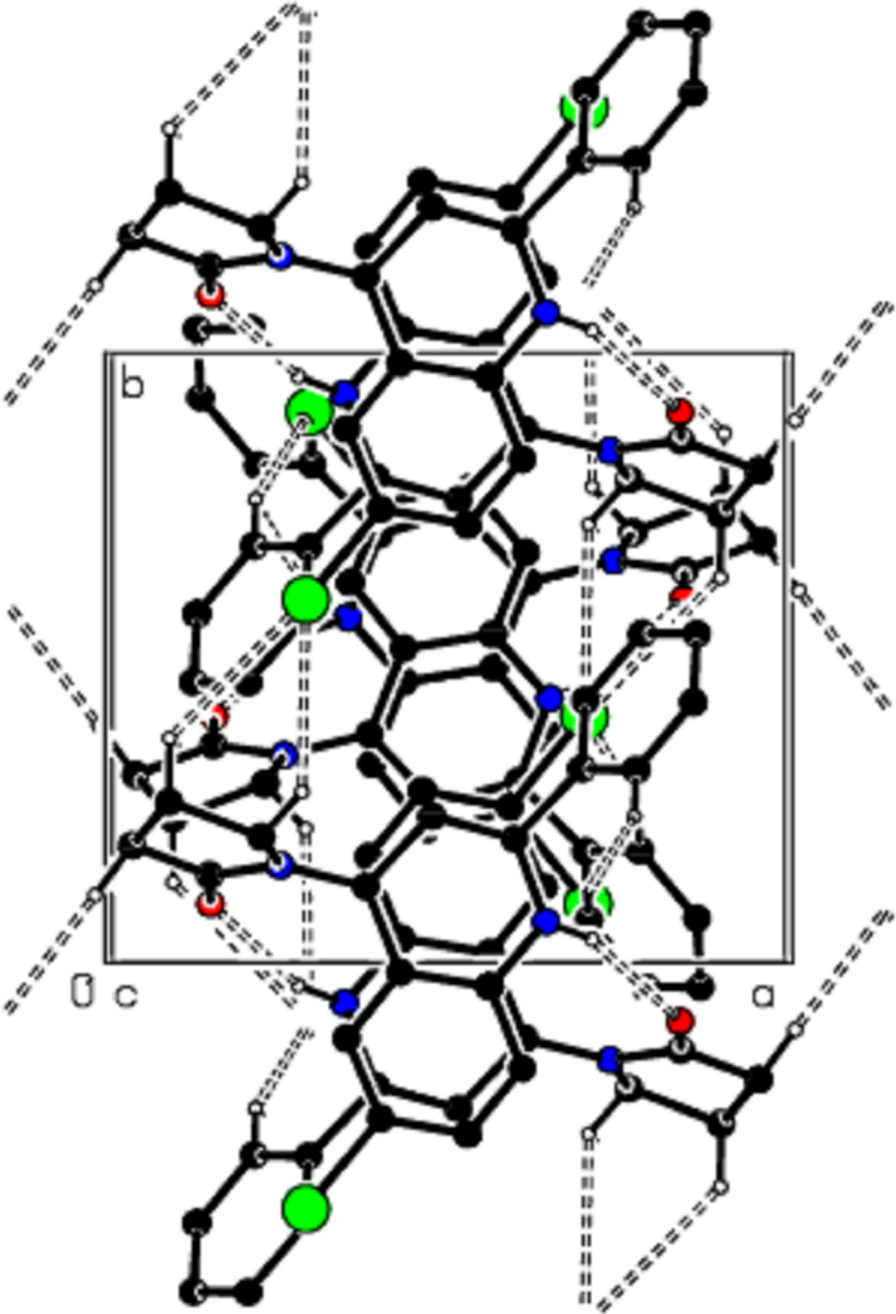
A view of the mol­ecular packing along the *c* axis of the title compound.

**Figure 7:**
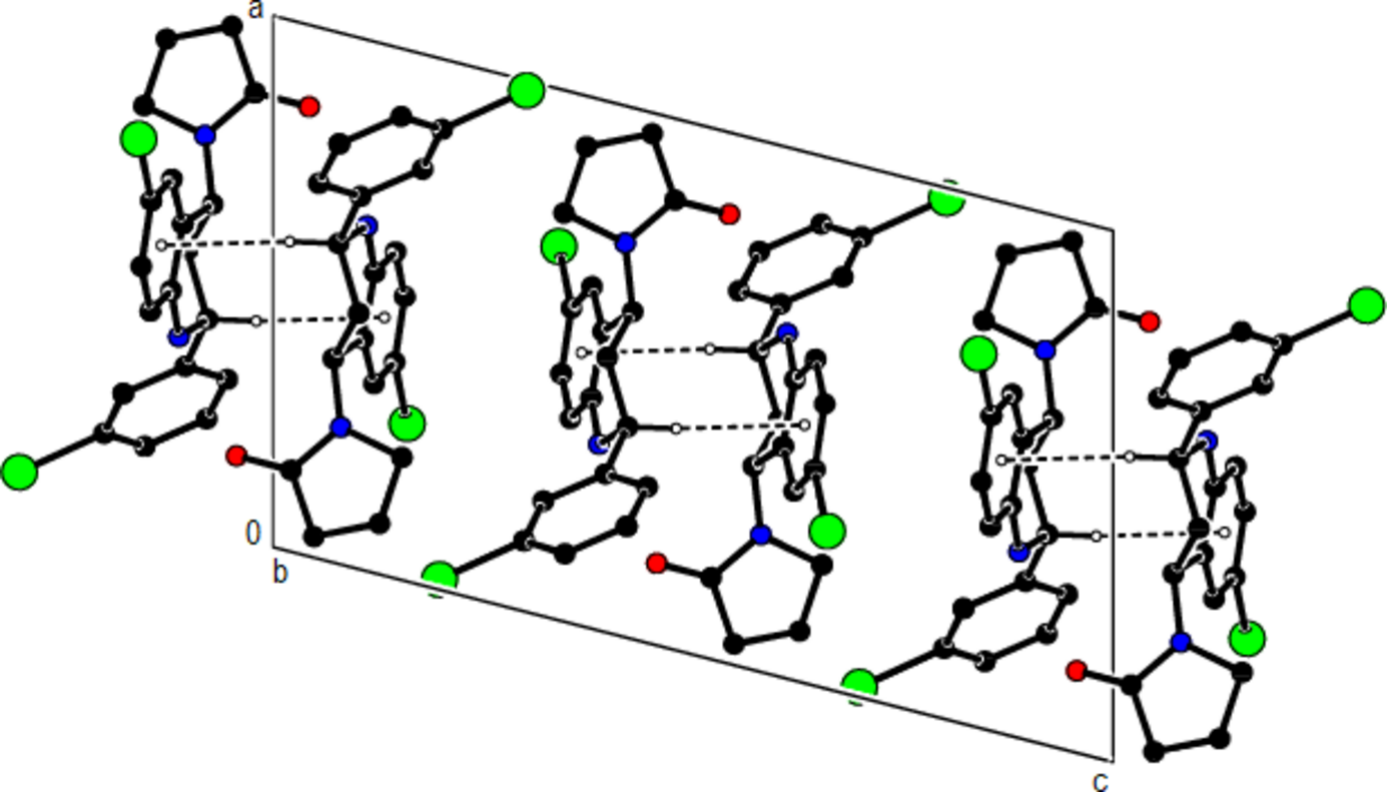
A view of the mol­ecular packing along the *b* axis of the title compound.

**Figure 8:**
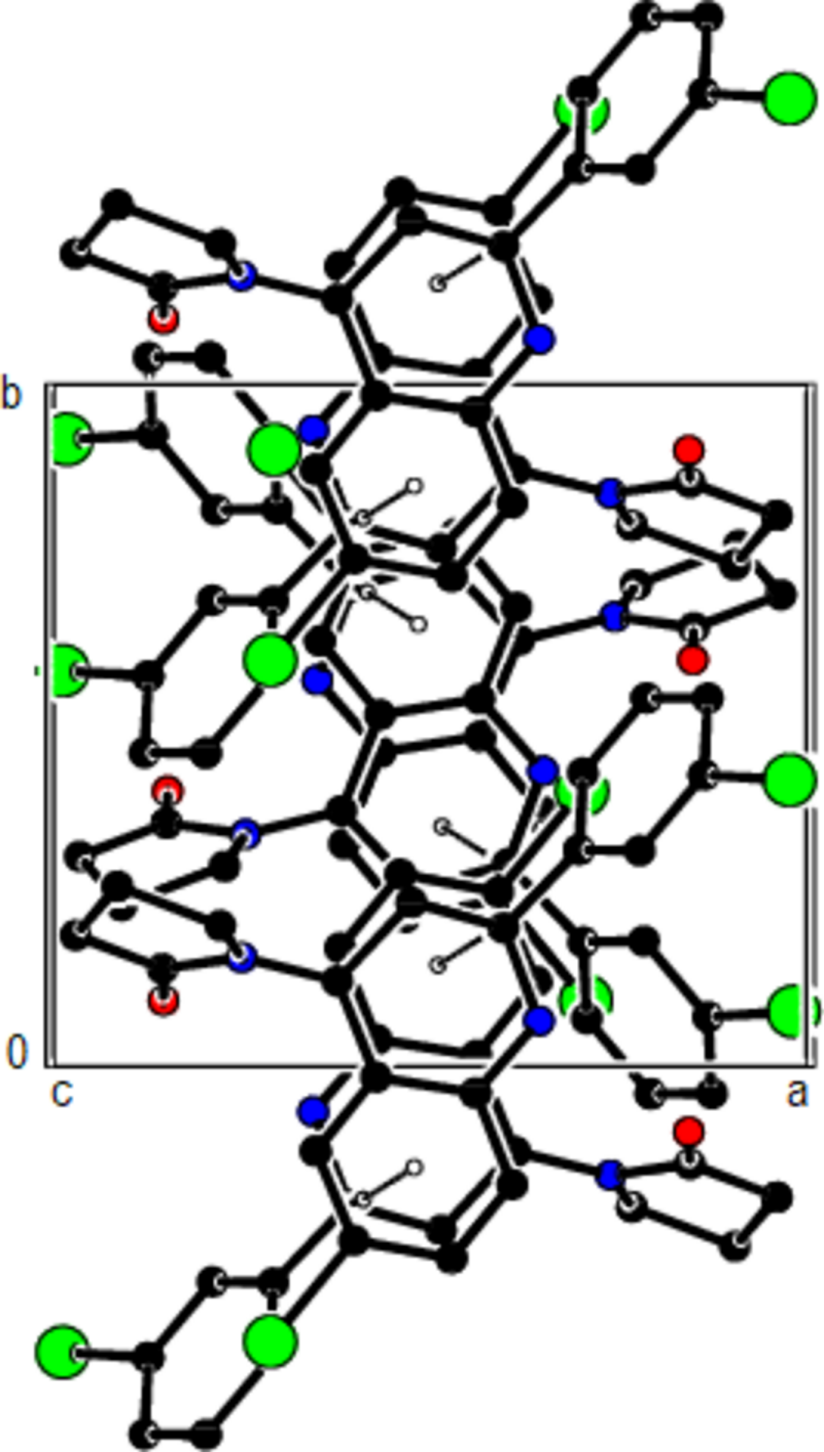
A view of the mol­ecular packing along the *c* axis of the title compound.

**Table 1:** Hydrogen-bond geometry (Å, °) *Cg*3 is the centroid of the C4*A*/C5–C8/C8*A* benzene ring.

*D*—H⋯*A*	*D*—H	H⋯*A*	*D*⋯*A*	*D*—H⋯*A*
C13—H13*A*⋯O1^i^	0.99	2.46	3.431 (3)	169
C13—H13*A*⋯Br2^ii^	0.99	3.06	3.740 (3)	127
C14—H14*A*⋯Br2^ii^	0.99	3.12	3.664 (3)	116
C14—H14*B*⋯Br1^iii^	0.99	3.02	3.786 (2)	135
C15—H15*A*⋯Br1^iii^	0.99	3.00	3.748 (2)	133
C15—H15*B*⋯Br2^ii^	0.99	3.09	3.768 (2)	127
C22—H22⋯Br1^iv^	0.95	2.94	3.820 (2)	155
C24—H24⋯Br2^v^	0.95	3.04	3.932 (2)	158
N1—H1⋯O1^vi^	0.86 (3)	2.30 (3)	3.115 (3)	159 (2)
C2—H2⋯*Cg*3^vi^	1.00	2.66	3.655 (3)	173

Symmetry codes: (i) 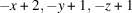; (ii) 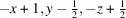; (iii) 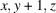; (iv) 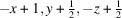; (v) 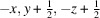; (vi) 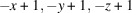.

**Table 2:** Experimental details

Crystal data	
Chemical formula	C_19_H_18_Br_2_N_2_O
*M*_r_	450.17
Crystal system, space group	Monoclinic, *P*2_1_/*c*
Temperature (K)	100
*a*, *b*, *c* (Å)	10.8691 (8), 9.4578 (7), 17.7217 (14)
β (°)	104.364 (3)
*V* (Å^3^)	1764.8 (2)
*Z*	4
Radiation type	Mo *K*α
μ (mm^−1^)	4.60
Crystal size (mm)	0.36 × 0.32 × 0.26

Data collection	
Diffractometer	Bruker Kappa APEXII area-detector
Absorption correction	Multi-scan (*SADABS*; Krause *et al.*, 2015 ▸)
*T*_min_, *T*_max_	0.714, 1.000
No. of measured, independent and observed [*I* > 2σ(*I*)] reflections	25906, 4041, 3231
*R*_int_	0.054
(sin θ/λ)_max_ (Å^−1^)	0.650

Refinement	
*R*[*F*^2^ > 2σ(*F*^2^)], *wR*(*F*^2^), *S*	0.029, 0.057, 1.02
No. of reflections	4041
No. of parameters	220
H-atom treatment	H atoms treated by a mixture of independent and constrained refinement
Δρ_max_, Δρ_min_ (e Å^−3^)	0.47, −0.47

Computer programs: *APEX3* and *SAINT* (Bruker, 2018 ▸), *SHELXT2014/5* (Sheldrick, 2015*a* ▸), *SHELXL2018/3* (Sheldrick, 2015*b* ▸), *ORTEP-3 for Windows* (Farrugia, 2012 ▸) and *PLATON* (Spek, 2020 ▸).

## supplementary crystallographic information

### 1-[6-Bromo-2-(3-bromophenyl)-1,2,3,4-tetrahydroquinolin-4-yl]pyrrolidin-2-one Crystal data

**Table:**

C_19_H_18_Br_2_N_2_O	*F*(000) = 896
*M**_r_* = 450.17	*D*_x_ = 1.694 Mg m^−^^3^
Monoclinic, *P*2_1_/*c*	Mo *K*α radiation, λ = 0.71073 Å
*a* = 10.8691 (8) Å	Cell parameters from 5029 reflections
*b* = 9.4578 (7) Å	θ = 2.9–26.4°
*c* = 17.7217 (14) Å	µ = 4.60 mm^−^^1^
β = 104.364 (3)°	*T* = 100 K
*V* = 1764.8 (2) Å^3^	Bulk, colourless
*Z* = 4	0.36 × 0.32 × 0.26 mm

### 1-[6-Bromo-2-(3-bromophenyl)-1,2,3,4-tetrahydroquinolin-4-yl]pyrrolidin-2-one Data collection

**Table:**

Bruker Kappa APEXII area-detector diffractometer	3231 reflections with *I* > 2σ(*I*)
φ and ω scans	*R*_int_ = 0.054
Absorption correction: multi-scan (SADABS; Krause *et al.*, 2015)	θ_max_ = 27.5°, θ_min_ = 4.2°
*T*_min_ = 0.714, *T*_max_ = 1.000	*h* = −13→14
25906 measured reflections	*k* = −11→12
4041 independent reflections	*l* = −23→23

### 1-[6-Bromo-2-(3-bromophenyl)-1,2,3,4-tetrahydroquinolin-4-yl]pyrrolidin-2-one Refinement

**Table:**

Refinement on *F*^2^	Primary atom site location: structure-invariant direct methods
Least-squares matrix: full	Hydrogen site location: mixed
*R*[*F*^2^ > 2σ(*F*^2^)] = 0.029	H atoms treated by a mixture of independent and constrained refinement
*wR*(*F*^2^) = 0.057	*w* = 1/[σ^2^(*F*_o_^2^) + (0.0176*P*)^2^ + 1.5651*P*] where *P* = (*F*_o_^2^ + 2*F*_c_^2^)/3
*S* = 1.01	(Δ/σ)_max_ = 0.001
4041 reflections	Δρ_max_ = 0.47 e Å^−^^3^
220 parameters	Δρ_min_ = −0.47 e Å^−^^3^
0 restraints	

### 1-[6-Bromo-2-(3-bromophenyl)-1,2,3,4-tetrahydroquinolin-4-yl]pyrrolidin-2-one Special details

**Table:**

Geometry. All esds (except the esd in the dihedral angle between two l.s. planes) are estimated using the full covariance matrix. The cell esds are taken into account individually in the estimation of esds in distances, angles and torsion angles; correlations between esds in cell parameters are only used when they are defined by crystal symmetry. An approximate (isotropic) treatment of cell esds is used for estimating esds involving l.s. planes.

### 1-[6-Bromo-2-(3-bromophenyl)-1,2,3,4-tetrahydroquinolin-4-yl]pyrrolidin-2-one Fractional atomic coordinates and isotropic or equivalent isotropic displacement parameters (Å^2^)

**Table:**

	*x*	*y*	*z*	*U*_iso_*/*U*_eq_	
C2	0.3972 (2)	0.7031 (2)	0.42197 (14)	0.0095 (5)	
H2	0.417106	0.695439	0.480026	0.011*	
C3	0.5184 (2)	0.7407 (2)	0.39791 (14)	0.0103 (5)	
H3A	0.551079	0.833284	0.420260	0.012*	
H3B	0.500982	0.746994	0.340468	0.012*	
C4	0.6161 (2)	0.6251 (2)	0.42821 (14)	0.0090 (5)	
H4A	0.623691	0.617392	0.485461	0.011*	
C4A	0.5649 (2)	0.4840 (2)	0.39280 (14)	0.0103 (5)	
C5	0.6438 (2)	0.3756 (2)	0.37979 (14)	0.0116 (5)	
H5A	0.732382	0.391768	0.387756	0.014*	
C6	0.5933 (2)	0.2444 (2)	0.35526 (14)	0.0141 (5)	
C7	0.4649 (2)	0.2184 (2)	0.34323 (14)	0.0153 (5)	
H7A	0.431556	0.127113	0.327685	0.018*	
C8	0.3852 (2)	0.3264 (2)	0.35403 (14)	0.0141 (5)	
H8A	0.296550	0.309470	0.344583	0.017*	
C8A	0.4337 (2)	0.4609 (2)	0.37876 (14)	0.0099 (5)	
C12	0.8464 (2)	0.6433 (2)	0.47857 (15)	0.0108 (5)	
C13	0.9605 (2)	0.6920 (2)	0.45130 (16)	0.0164 (6)	
H13A	1.014249	0.610573	0.444761	0.020*	
H13B	1.012448	0.759132	0.488933	0.020*	
C14	0.9045 (2)	0.7642 (2)	0.37321 (16)	0.0183 (6)	
H14A	0.954217	0.740510	0.335083	0.022*	
H14B	0.903707	0.868151	0.379516	0.022*	
C15	0.7689 (2)	0.7064 (2)	0.34649 (15)	0.0140 (5)	
H15A	0.708860	0.781053	0.321194	0.017*	
H15B	0.764491	0.626563	0.309794	0.017*	
C21	0.2979 (2)	0.8177 (2)	0.39562 (14)	0.0098 (5)	
C22	0.2170 (2)	0.8162 (2)	0.32186 (14)	0.0108 (5)	
H22	0.219100	0.739884	0.287317	0.013*	
C23	0.1327 (2)	0.9274 (2)	0.29887 (14)	0.0113 (5)	
C24	0.1280 (2)	1.0400 (2)	0.34733 (16)	0.0176 (6)	
H24	0.070147	1.115604	0.330570	0.021*	
C25	0.2095 (3)	1.0404 (3)	0.42109 (17)	0.0207 (6)	
H25	0.207518	1.117252	0.455296	0.025*	
C26	0.2935 (2)	0.9304 (2)	0.44550 (15)	0.0157 (5)	
H26	0.348384	0.931566	0.496389	0.019*	
N1	0.35049 (19)	0.5666 (2)	0.38807 (12)	0.0121 (4)	
H1	0.283 (3)	0.538 (3)	0.3999 (15)	0.014*	
N11	0.74188 (18)	0.65967 (19)	0.41920 (12)	0.0105 (4)	
O1	0.84580 (15)	0.59639 (17)	0.54316 (10)	0.0153 (4)	
Br1	0.70318 (3)	0.09579 (2)	0.34029 (2)	0.02171 (8)	
Br2	0.01897 (2)	0.91972 (2)	0.19816 (2)	0.01641 (7)	

### 1-[6-Bromo-2-(3-bromophenyl)-1,2,3,4-tetrahydroquinolin-4-yl]pyrrolidin-2-one Atomic displacement parameters (Å^2^)

**Table:**

	*U*^11^	*U*^22^	*U*^33^	*U*^12^	*U*^13^	*U*^23^
C2	0.0097 (12)	0.0106 (11)	0.0073 (13)	0.0004 (9)	0.0003 (9)	0.0003 (9)
C3	0.0121 (12)	0.0083 (10)	0.0106 (13)	0.0009 (9)	0.0031 (10)	0.0009 (9)
C4	0.0072 (11)	0.0102 (10)	0.0097 (13)	0.0008 (8)	0.0025 (9)	0.0018 (9)
C4A	0.0143 (13)	0.0078 (10)	0.0088 (13)	0.0014 (9)	0.0031 (10)	0.0028 (9)
C5	0.0144 (13)	0.0126 (11)	0.0092 (13)	0.0020 (9)	0.0052 (10)	0.0031 (9)
C6	0.0222 (14)	0.0103 (11)	0.0114 (13)	0.0063 (10)	0.0073 (11)	0.0023 (10)
C7	0.0257 (15)	0.0089 (11)	0.0098 (14)	−0.0016 (10)	0.0016 (11)	−0.0014 (9)
C8	0.0156 (13)	0.0135 (11)	0.0120 (14)	−0.0024 (10)	0.0011 (10)	0.0017 (10)
C8A	0.0132 (13)	0.0106 (10)	0.0059 (12)	0.0003 (9)	0.0021 (10)	0.0031 (9)
C12	0.0106 (12)	0.0065 (10)	0.0149 (14)	0.0024 (9)	0.0023 (10)	−0.0036 (9)
C13	0.0094 (13)	0.0146 (12)	0.0248 (16)	0.0010 (9)	0.0034 (11)	−0.0036 (10)
C14	0.0140 (13)	0.0136 (12)	0.0302 (17)	0.0008 (10)	0.0111 (12)	0.0043 (11)
C15	0.0152 (13)	0.0117 (11)	0.0170 (14)	0.0037 (9)	0.0074 (11)	0.0025 (10)
C21	0.0079 (12)	0.0109 (11)	0.0117 (13)	−0.0005 (9)	0.0047 (10)	0.0021 (9)
C22	0.0110 (12)	0.0115 (11)	0.0107 (13)	−0.0001 (9)	0.0044 (10)	−0.0009 (9)
C23	0.0081 (11)	0.0128 (11)	0.0117 (13)	−0.0021 (9)	0.0003 (9)	0.0032 (10)
C24	0.0131 (13)	0.0112 (11)	0.0283 (17)	0.0045 (9)	0.0047 (11)	0.0039 (10)
C25	0.0245 (15)	0.0128 (12)	0.0253 (16)	0.0015 (10)	0.0074 (12)	−0.0068 (11)
C26	0.0163 (13)	0.0188 (12)	0.0098 (13)	−0.0004 (10)	−0.0009 (10)	−0.0013 (10)
N1	0.0070 (10)	0.0101 (9)	0.0194 (12)	−0.0008 (8)	0.0036 (9)	−0.0001 (8)
N11	0.0100 (10)	0.0113 (9)	0.0106 (11)	0.0010 (8)	0.0030 (8)	0.0012 (8)
O1	0.0141 (9)	0.0190 (9)	0.0112 (9)	0.0034 (7)	0.0001 (7)	−0.0003 (7)
Br1	0.03536 (17)	0.01082 (12)	0.02535 (16)	0.00804 (11)	0.01967 (13)	0.00283 (11)
Br2	0.01226 (13)	0.01562 (12)	0.01764 (15)	−0.00140 (10)	−0.00331 (10)	0.00727 (10)

### 1-[6-Bromo-2-(3-bromophenyl)-1,2,3,4-tetrahydroquinolin-4-yl]pyrrolidin-2-one Geometric parameters (Å, º)

**Table:**

C2—N1	1.460 (3)	C12—C13	1.511 (3)
C2—C21	1.519 (3)	C13—C14	1.528 (4)
C2—C3	1.524 (3)	C13—H13A	0.9900
C2—H2	1.0000	C13—H13B	0.9900
C3—C4	1.526 (3)	C14—C15	1.532 (3)
C3—H3A	0.9900	C14—H14A	0.9900
C3—H3B	0.9900	C14—H14B	0.9900
C4—N11	1.453 (3)	C15—N11	1.459 (3)
C4—C4A	1.520 (3)	C15—H15A	0.9900
C4—H4A	1.0000	C15—H15B	0.9900
C4A—C5	1.392 (3)	C21—C22	1.383 (3)
C4A—C8A	1.402 (3)	C21—C26	1.393 (3)
C5—C6	1.383 (3)	C22—C23	1.389 (3)
C5—H5A	0.9500	C22—H22	0.9500
C6—C7	1.380 (4)	C23—C24	1.376 (3)
C6—Br1	1.905 (2)	C23—Br2	1.903 (2)
C7—C8	1.383 (3)	C24—C25	1.385 (4)
C7—H7A	0.9500	C24—H24	0.9500
C8—C8A	1.404 (3)	C25—C26	1.381 (3)
C8—H8A	0.9500	C25—H25	0.9500
C8A—N1	1.386 (3)	C26—H26	0.9500
C12—O1	1.229 (3)	N1—H1	0.86 (3)
C12—N11	1.352 (3)		
			
N1—C2—C21	110.84 (19)	C14—C13—H13A	110.8
N1—C2—C3	109.13 (19)	C12—C13—H13B	110.8
C21—C2—C3	110.18 (18)	C14—C13—H13B	110.8
N1—C2—H2	108.9	H13A—C13—H13B	108.9
C21—C2—H2	108.9	C13—C14—C15	104.92 (19)
C3—C2—H2	108.9	C13—C14—H14A	110.8
C2—C3—C4	107.97 (18)	C15—C14—H14A	110.8
C2—C3—H3A	110.1	C13—C14—H14B	110.8
C4—C3—H3A	110.1	C15—C14—H14B	110.8
C2—C3—H3B	110.1	H14A—C14—H14B	108.8
C4—C3—H3B	110.1	N11—C15—C14	102.9 (2)
H3A—C3—H3B	108.4	N11—C15—H15A	111.2
N11—C4—C4A	114.22 (19)	C14—C15—H15A	111.2
N11—C4—C3	113.07 (18)	N11—C15—H15B	111.2
C4A—C4—C3	109.42 (19)	C14—C15—H15B	111.2
N11—C4—H4A	106.5	H15A—C15—H15B	109.1
C4A—C4—H4A	106.5	C22—C21—C26	119.6 (2)
C3—C4—H4A	106.5	C22—C21—C2	121.3 (2)
C5—C4A—C8A	119.8 (2)	C26—C21—C2	119.0 (2)
C5—C4A—C4	122.5 (2)	C21—C22—C23	119.2 (2)
C8A—C4A—C4	117.5 (2)	C21—C22—H22	120.4
C6—C5—C4A	120.0 (2)	C23—C22—H22	120.4
C6—C5—H5A	120.0	C24—C23—C22	121.8 (2)
C4A—C5—H5A	120.0	C24—C23—Br2	119.92 (18)
C7—C6—C5	121.1 (2)	C22—C23—Br2	118.28 (18)
C7—C6—Br1	119.47 (17)	C23—C24—C25	118.5 (2)
C5—C6—Br1	119.45 (19)	C23—C24—H24	120.7
C6—C7—C8	119.4 (2)	C25—C24—H24	120.7
C6—C7—H7A	120.3	C26—C25—C24	120.8 (2)
C8—C7—H7A	120.3	C26—C25—H25	119.6
C7—C8—C8A	120.9 (2)	C24—C25—H25	119.6
C7—C8—H8A	119.6	C25—C26—C21	120.1 (2)
C8A—C8—H8A	119.6	C25—C26—H26	119.9
N1—C8A—C4A	122.1 (2)	C21—C26—H26	119.9
N1—C8A—C8	119.0 (2)	C8A—N1—C2	121.08 (19)
C4A—C8A—C8	118.9 (2)	C8A—N1—H1	115.4 (17)
O1—C12—N11	124.7 (2)	C2—N1—H1	114.1 (17)
O1—C12—C13	127.0 (2)	C12—N11—C4	121.4 (2)
N11—C12—C13	108.3 (2)	C12—N11—C15	114.0 (2)
C12—C13—C14	104.5 (2)	C4—N11—C15	124.49 (19)
C12—C13—H13A	110.8		
			
N1—C2—C3—C4	−60.0 (2)	N1—C2—C21—C26	149.1 (2)
C21—C2—C3—C4	178.05 (19)	C3—C2—C21—C26	−90.0 (3)
C2—C3—C4—N11	−171.22 (19)	C26—C21—C22—C23	0.0 (3)
C2—C3—C4—C4A	60.2 (2)	C2—C21—C22—C23	−176.4 (2)
N11—C4—C4A—C5	22.9 (3)	C21—C22—C23—C24	0.5 (4)
C3—C4—C4A—C5	150.8 (2)	C21—C22—C23—Br2	−178.16 (17)
N11—C4—C4A—C8A	−162.0 (2)	C22—C23—C24—C25	−0.5 (4)
C3—C4—C4A—C8A	−34.1 (3)	Br2—C23—C24—C25	178.1 (2)
C8A—C4A—C5—C6	−1.9 (4)	C23—C24—C25—C26	0.0 (4)
C4—C4A—C5—C6	173.1 (2)	C24—C25—C26—C21	0.5 (4)
C4A—C5—C6—C7	0.0 (4)	C22—C21—C26—C25	−0.5 (4)
C4A—C5—C6—Br1	−178.40 (18)	C2—C21—C26—C25	176.0 (2)
C5—C6—C7—C8	1.6 (4)	C4A—C8A—N1—C2	−7.4 (4)
Br1—C6—C7—C8	−179.91 (18)	C8—C8A—N1—C2	173.0 (2)
C6—C7—C8—C8A	−1.5 (4)	C21—C2—N1—C8A	155.7 (2)
C5—C4A—C8A—N1	−177.6 (2)	C3—C2—N1—C8A	34.2 (3)
C4—C4A—C8A—N1	7.2 (3)	O1—C12—N11—C4	1.7 (3)
C5—C4A—C8A—C8	2.0 (3)	C13—C12—N11—C4	−178.46 (19)
C4—C4A—C8A—C8	−173.2 (2)	O1—C12—N11—C15	−175.2 (2)
C7—C8—C8A—N1	179.3 (2)	C13—C12—N11—C15	4.7 (3)
C7—C8—C8A—C4A	−0.3 (4)	C4A—C4—N11—C12	−101.7 (2)
O1—C12—C13—C14	−169.8 (2)	C3—C4—N11—C12	132.3 (2)
N11—C12—C13—C14	10.3 (2)	C4A—C4—N11—C15	74.8 (3)
C12—C13—C14—C15	−20.3 (2)	C3—C4—N11—C15	−51.2 (3)
C13—C14—C15—N11	22.5 (2)	C14—C15—N11—C12	−17.5 (2)
N1—C2—C21—C22	−34.5 (3)	C14—C15—N11—C4	165.80 (19)
C3—C2—C21—C22	86.4 (3)		

### 1-[6-Bromo-2-(3-bromophenyl)-1,2,3,4-tetrahydroquinolin-4-yl]pyrrolidin-2-one Hydrogen-bond geometry (Å, º)

*Cg*3 is the centroid of the C4*A*/C5–C8/C8*A* benzene ring.

**Table:**

*D*—H···*A*	*D*—H	H···*A*	*D*···*A*	*D*—H···*A*
C13—H13*A*···O1^i^	0.99	2.46	3.431 (3)	169
C13—H13*A*···Br2^ii^	0.99	3.06	3.740 (3)	127
C14—H14*A*···Br2^ii^	0.99	3.12	3.664 (3)	116
C14—H14*B*···Br1^iii^	0.99	3.02	3.786 (2)	135
C15—H15*A*···Br1^iii^	0.99	3.00	3.748 (2)	133
C15—H15*B*···Br2^ii^	0.99	3.09	3.768 (2)	127
C22—H22···Br1^iv^	0.95	2.94	3.820 (2)	155
C24—H24···Br2^v^	0.95	3.04	3.932 (2)	158
N1—H1···O1^vi^	0.86 (3)	2.30 (3)	3.115 (3)	159 (2)
C2—H2···*Cg*3^vi^	1.00	2.66	3.655 (3)	173

Symmetry codes: (i) −*x*+2, −*y*+1, −*z*+1; (ii) −*x*+1, *y*−1/2, −*z*+1/2; (iii) *x*, *y*+1, *z*; (iv) −*x*+1, *y*+1/2, −*z*+1/2; (v) −*x*, *y*+1/2, −*z*+1/2; (vi) −*x*+1, −*y*+1, −*z*+1.
